# Gper1 inhibition exacerbates traumatic brain injury-induced neurological impairments in mice

**DOI:** 10.1186/s12993-025-00281-2

**Published:** 2025-07-02

**Authors:** Ya-Fei Xue, Ying-Xi Wu, Yun-Ze Zhang, Tian-Zhi Zhao

**Affiliations:** Department of Neurosurgery, the Second Affiliated Hospital of Air Force Medical University, No.569 Xinsi Road, Xi’an, 710038 Shaanxi China

**Keywords:** Traumatic brain injury, G protein-coupled Estrogen receptor 1, Neuroinflammation, Impairments

## Abstract

**Background:**

G protein-coupled estrogen receptor 1 (Gper1) is widely expressed in the brain, while its function in traumatic brain injury (TBI) remains poorly understood. This study aims to investigate the role of Gper1 in TBI pathology and the underlying mechanisms using a mouse model.

**Methods:**

Gper1 knockout (*Gper1*^*KO*^) mice were generated, and TBI was induced via controlled cortical impact (CCI). Brain water content, cell apoptosis, and neuroinflammation were assessed using real-time polymerase chain reaction, enzyme-linked immunosorbent assay, and TUNEL staining. Behavioral outcomes, including cognitive and anxiety-related behaviors, were evaluated using the open field test and Y-maze test.

**Results:**

Gper1 expression was significantly upregulated in the brain tissues of TBI mice. Knockout of *Gper1* led to exacerbated TBI-induced outcomes, including increased brain edema, blood-brain barrier disruption, and aggravated cell apoptosis and neuroinflammation in the cortex. Behaviorally, *Gper1*^*KO*^ mice displayed more severe cognitive impairments and anxiety-like behaviors compared to wild-type mice.

**Conclusions:**

Gper1 inhibition exacerbates TBI-induced neurological and behavioral impairments, which suggests that Gper1 may be a potential therapeutic target for mitigating TBI-associated brain injury.

**Supplementary Information:**

The online version contains supplementary material available at 10.1186/s12993-025-00281-2.

## Introduction

Traumatic brain injury (TBI) is defined as the destruction of brain tissue structure or dysfunction caused by external force on the head [1]. TBI has become the leading cause of death and disability among all trauma-related injuries worldwide [2]. A large data analysis covering 16 countries in Europe showed that the number of people admitted to hospital due to TBI was 1287.2/100,000, and the fatality rate was 11.7% [3]. In recent years, the mortality rate of TBI has declined with the establishment of a professional Intensive Care Unit and the implementation of evidence-based guidelines for TBI [4]. However, in a large number of surviving TBI patients, there are still multiple neurological functional impairments caused by neuronal damage, including impaired thinking, language, learning, emotional and behavioral cognitive dysfunction, and even mental disorders [5]. The mechanisms of neuronal injury after TBI are complex, and the molecular targets involved in the process have not yet been clarified [6]. Therefore, it is extremely important to understand the pathological process, which is critical for the treatment of TBI.

G protein-coupled estrogen receptor 1 (GPER1) is a seven-transmembrane receptor consisting of 375 amino acids [7]. As an estrogen membrane receptor, GPER1 mediates estrogen nongenomic biological effects in neurons [8]. It has been observed that GPER1 is widely expressed in the brain, especially in the hypothalamus-pituitary axis, hippocampus, and brainstem autonomic nucleus [9]. In recent years, it is revealed that GPER1 not only exists in the brain, but also exerts physiological functions [10]. It has been found that the GPER1 agonist G1 can increase the excitatory postsynaptic currents of vertebral cells in the hippocampal slices of ovariectomized female rats [11]. The results from in vitro experiments suggest that GPER1 can promote the release of calcium in neurons and activate the mitogen-activated protein kinase (MAPK) signaling pathway. At the same time, it has been demonstrated that GPER1 has a protective effect on trauma-induced organ damage, which is related to the activation of protein kinase A signaling pathway in cells by GPER1 [12].

Our previous work demonstrates that GPER1 agonists alleviates TBI-induced cognitive impairment and brain edema in mice by regulating microglia-mediated neuroinflammation [13]. The purpose of this work is to continue to explore the role and mechanisms of GPER1 in TBI, and to study the effects of *Gper1* knockout on brain edema, cognitive function, cell apoptosis and neuroinflammation in mice.

## Methods

### Animals

The protocol was approved by the ethics committee of Tangdu Hospital (#20241199). *Gper1* conditional knockout mice (*Gper1*^KO^) were purchased from Saiye Biotech (Suzhou, China). The strain name is C57BL/6J-Gper1em1Cya, and the strain number is KOCMP-76,854-Gper1-B6J-VA. The genetic background of the strain is C57BL/6J. A total of 62 wild type mice and 32 *Gper1*^*KO*^ mice were used in this study. Cryopreserved strains of mouse sperm were purchased. Homozygous *Gper1*^KO^ mice were obtained after expansion and screening. The wild type mice in this study are C57BL/6J mice. All mice were housed in a 12-hour cycle of alternating light and dark. Mice were housed at 22 ± 2 °C and 50 ± 1% relative humidity. Sterile food and drinking water were freely available. Eight-week-old female C57BL/6J mice and *Gper1*^KO^ mice were selected for this study. Before experimental mice underwent TBI modeling, they first underwent bilateral ovariectomy with bilateral dorsolateral incisions in the abdominal frontal wall.

The study included two experimental groups: Wild-type (WT) Group: C57BL/6J mice with intact *Gper1* expression. *Gper1*^*KO*^ Group: Homozygous Gper1 knockout mice with Gper1 function completely ablated. Both groups were subjected to the same experimental procedures, including TBI modeling, behavioral tests, and molecular/biochemical analyses, as outlined in the methods section.

### Controlled Cortical Impact (CCI)

Unilateral CCI was used to simulate TBI in mice. Mice previously anesthetized with isoflurane were head-fixed in a transparent rigid positioning frame. Trained laboratory personnel used sterile equipment in a sterile operating table to open the mouse skin to expose the skull and bregma. Sterile drills for mouse bones were used for the parietal lobe craniotomy. The diameter of the opening was 3.5 mm. The opening was centered 0.5 mm anterior and 2.0 mm lateral to the middle bregma. The mouse’s dura mater and cerebral cortex were exposed. The CCI instrument applied in this study was pneumatically driven and the diameter of the impactor was 3 mm. Each impact could compress the mouse’s brain tissue to 1.5 millimeters. The maximum impact velocity was 3.75 m/s. The residence time of each compression shock head in the mouse brain was 150 ms. After surgery, the skin of the mouse brain was sutured. Mice were maintained under sterile conditions. The control group only received the same anesthesia and brain skin incision as the TBI group.

### Blood Brain Barrier (BBB) dysfunction

Brain tissue water content was used to evaluate the BBB dysfunction in this study. The wet/dry method was used to detect the water content of brain tissue on the third day after TBI modeling. Immediately after the animal was sacrificed, the brain tissue was removed and sliced into 4 mm thick brain slices. Electronic analytical balance weighed each part, i.e. wet weight (WW). Then the brain slices were placed at 100 °C for 24 h and weighed, that was dry weight (DW). The formula [WW − DW]/WW×100% calculated the brain tissue water content (%).

### Evans blue extravasation assay

The Evans blue extravasation assay was used to evaluate BBB permeability following TBI. Mice were anesthetized with isoflurane and injected intravenously with 2% Evans blue dye (4 mL/kg body weight) via the tail vein. The dye was allowed to circulate for 2 h. After circulation, the mice were perfused transcardially with cold phosphate-buffered saline (PBS) to remove intravascular Evans blue. The brains were then carefully dissected, and the ipsilateral cortex was isolated, weighed, and homogenized in 1 mL of formamide. The homogenates were incubated at 55 °C for 24 h to extract the Evans blue dye. The samples were centrifuged at 12,000 × g for 30 min, and the absorbance of the supernatants was measured at 620 nm using a spectrophotometer. The concentration of Evans blue was calculated based on a standard curve and normalized to the weight of the tissue. Results were expressed as micrograms of Evans blue per gram of brain tissue (µg/g). This assay was performed in triplicate for each experimental group.

### TUNEL staining

Seven days after mice underwent TBI modeling, TUNEL staining was performed. Brains of euthanized mice were removed and fixed in 4% paraformaldehyde for 12 h. 30% sucrose was readily used for tissue dehydration. The mouse brains were sliced at 0.5 mm anterior to bregma. TUNEL staining was checked with the In Situ Cell Death Detection Kit (11684817910, Roche, Penzberg, Upper Bavaria, Germany). Images were taken using an Olympus fluorescence microscope, and the apoptosis index was calculated as the proportion of TUNEL-positive cells.

### Open Field Test (OFT)

Mice that underwent TBI modeling underwent OFT on day 7 post modeling. TBI mice and control mice were housed in 55 × 55 × 36 cm transparent rigid plastic boxes. Their movement trajectories were recorded and analyzed. Two days before the formal test, mice were placed in the open field box for 5 min each day (data not recorded) to gradually acclimate to the environment. Mice were sequentially placed in the center of the field, and their locomotor activity was recorded for 5 min. The site is divided into four quadrants with the same area and shape by the scale on the plastic plate. The area of each quadrant is 27.5 × 27.5 cm. The timing and frequency of activity of mice in different areas (central, extracentral and corner) in four quadrants were recorded. The distance, speed, and round-trip frequency of the mice were recorded and analyzed. Laboratory lighting, temperature, and humidity were kept constant. After each mouse was tested, the apparatus was wiped with 75% ethanol or an odorless cleaning agent to eliminate residual odors. All animal handling was performed by the same person to ensure consistent placement of the mice. Data recording was carried out by the same individual.

### Y maze test

The Y-maze test was conducted to assess spatial working memory and cognitive function in TBI and control mice, reflecting hippocampus-dependent cognitive performance. Before the Y-maze test, it was ensured that the mice had not been exposed to similar apparatuses to avoid learning effects influencing the results. On the seventh day after TBI modeling and following the open field test, mice were subjected to the Y-maze test. The Y-maze consists of three arms, with one arm closed during the training phase. Mice were initially allowed to explore the two open arms for 10 min, followed by a 30-minute rest in their home cages. During the test phase, all three arms were opened, and the mice were allowed to explore freely. Movements were recorded using the Y-maze system (Muromachi Kikai), and the percentage of new arm entries was calculated as a measure of spatial working memory. Higher alternation rates indicate better cognitive function, while lower rates suggest spatial memory impairments. Laboratory lighting, temperature, and humidity were kept constant. After each mouse was tested, the apparatus was wiped with 75% ethanol or an odorless cleaning agent to eliminate residual odors. All animal handling was performed by the same person to ensure consistent placement of the mice. Data recording was carried out by the same individual.

### Real-Time Polymerase Chain Reaction (RT-PCR)

SuperScript IV rt-PCR Kit (Thermo Fisher) was used to analyze gene expression according to the instructions. The relative expression of target genes was normalized by *Gapdh* and calculated by the 2^−ΔΔCT^ method.

*Gper1*:

Forward: ATGGATGCGACTACTCCAGC,

Reverse: AAGAGGGCAATCACGTACTGC;

*PGC-1α*

Forward: GAAAGGGCCAAACAGAGAGA,

Reverse: GTAAATCACACGGCGCTCTT;

*NLRP3*:

Forward: ATTACCCGCCCGAGAAAGG,

Reverse: CATGAGTGTGGCTAGATCCAAG;

*ASC*:

Forward: CTTGTCAGGGGATGAACTCAAAA,

Reverse: GCCATACGACTCCAGATAGTAGC;

*Gapdh*:

Forward: AATGGATTTGGACGCATTGGT,

Reverse: TTTGCACTGGTACGTGTTGAT.

### Western blot analysis

Western blotting was performed to evaluate the protein expression levels of PGC-1α, NLRP3, and ASC in the lesioned cortices post-TBI. Briefly, cortical tissues were homogenized in RIPA lysis buffer containing protease and phosphatase inhibitors. Protein concentrations were quantified using the BCA assay. Equal amounts of protein (20–30 µg) were separated by SDS-PAGE and transferred onto PVDF membranes. The membranes were blocked with 5% non-fat milk in TBST (Tris-buffered saline with 0.1% Tween-20) for 1 h at room temperature and then incubated overnight at 4 °C with primary antibodies specific to PGC-1α, NLRP3, ASC, and GAPDH (used as a loading control). After washing, the membranes were incubated with HRP-conjugated secondary antibodies for 1 h at room temperature. Protein bands were visualized using enhanced chemiluminescence (ECL) and quantified with ImageJ software. The expression levels of target proteins were normalized to GAPDH and compared to the WT-Sham group. Each group included three independent replicates, with tissue homogenates pooled from ten samples per group.

### Enzyme Linked Immunosorbent Assay (ELISA)

Mouse Interleukin 6 (IL-6) ELISA Kit (ab222503), Mouse IL-1 beta ELISA Kit (ab197742), Mouse Tumor necrosis factor (TNF) alpha ELISA Kit (ab208348) and Mouse Monocyte chemotactic protein 1 (MCP-1) ELISA Kit (ab208979) (Abcam, Cambridge, MA) were used for the detection of the neuroinflammation.

### Statistical analysis

Statistical software SPSS 24.0 was used to analyze the data. The measurement data was represented by mean ± standard deviation (SD). Shapiro-Wilk test was used to assess the normality of the data, and the results indicated that all data followed a normal distribution. The significances were analyzed by one-way ANOVA followed Dunn’s multiple comparisons test for the data set only involved the time variable, or two-way ANOVA followed Tukey’s multiple comparisons test for the data set involved two variables (genotype and TBI modeling status). *P* < 0.05 was considered statistically significant. For the results derived from multiple comparisons, we calculated the effect size using the formula: Cohen’s d = Mean Difference / SD, based on the mean differences and standard deviations between groups.

## Results

### Gper1 expression in the ipsilateral cortex in TBI mice

**Fig. 1:**
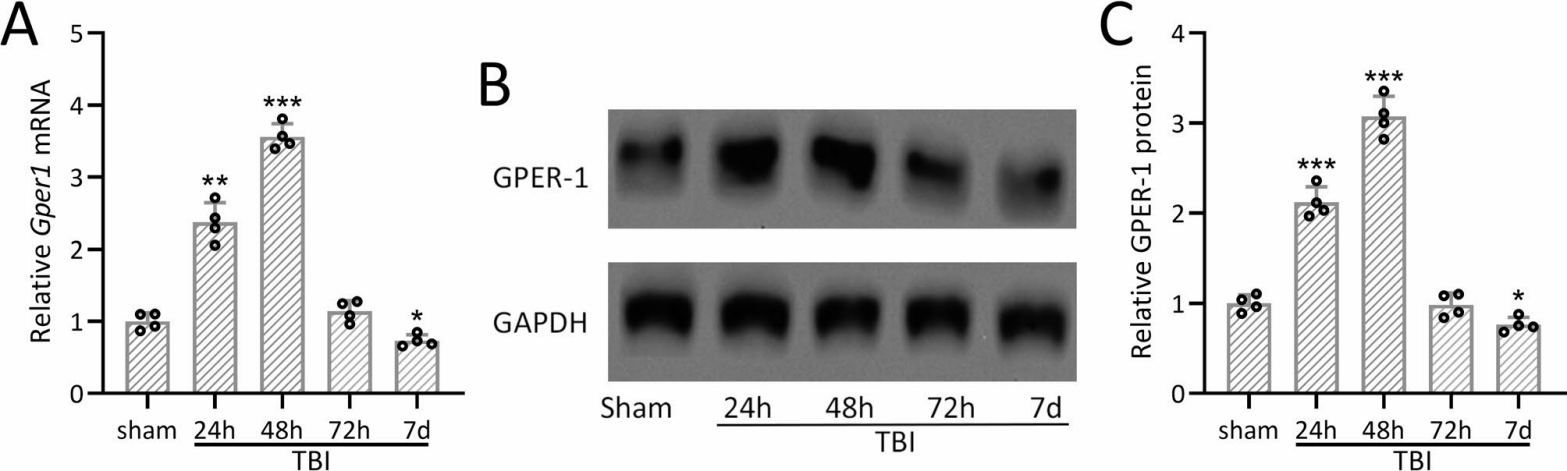
Gper1 expressions in the ipsilateral cortex after traumatic brain injury in wild type mice. qRT-PCR and western blotting were used to measure the mRNA (**A**) and proteins (**B** and **C**) levels of Gper1 in the ipsilateral cortex of wild type TBI mice. The expressions were normalized to sham. Data was shown with mean ± SD. *N* = 4 repeats for each group (6 tissue homogenates were mixed for each group). *p < 0.05, **p < 0.01, ***p < 0.001 compared to sham. One-way ANOVA followed Dunn’s multiple comparisons test

The totally experiment flow chart is shown in Figure S1. To illustrate the role of Gper1 in the development of TBI, we first explored the expression level of Gper1 in the brain tissues of TBI mice. As shown in Fig. 1A, the mRNA level of *Gper1* was significantly increased at 24 h after TBI modeling and was highest at 48 h. The mRNA of *Gper1* was significantly decreased at 72 h after TBI modeling. Similarly, the expression of Gper1 protein in mouse brain tissues was gradually increased at 24 and 48 h after brain injury, and was decreased at 72 h post-TBI (Fig. 1B and C).

### Gper1 knockout exacerbated TBI-induced brain edema and BBB dysfunction

**Fig. 2:**
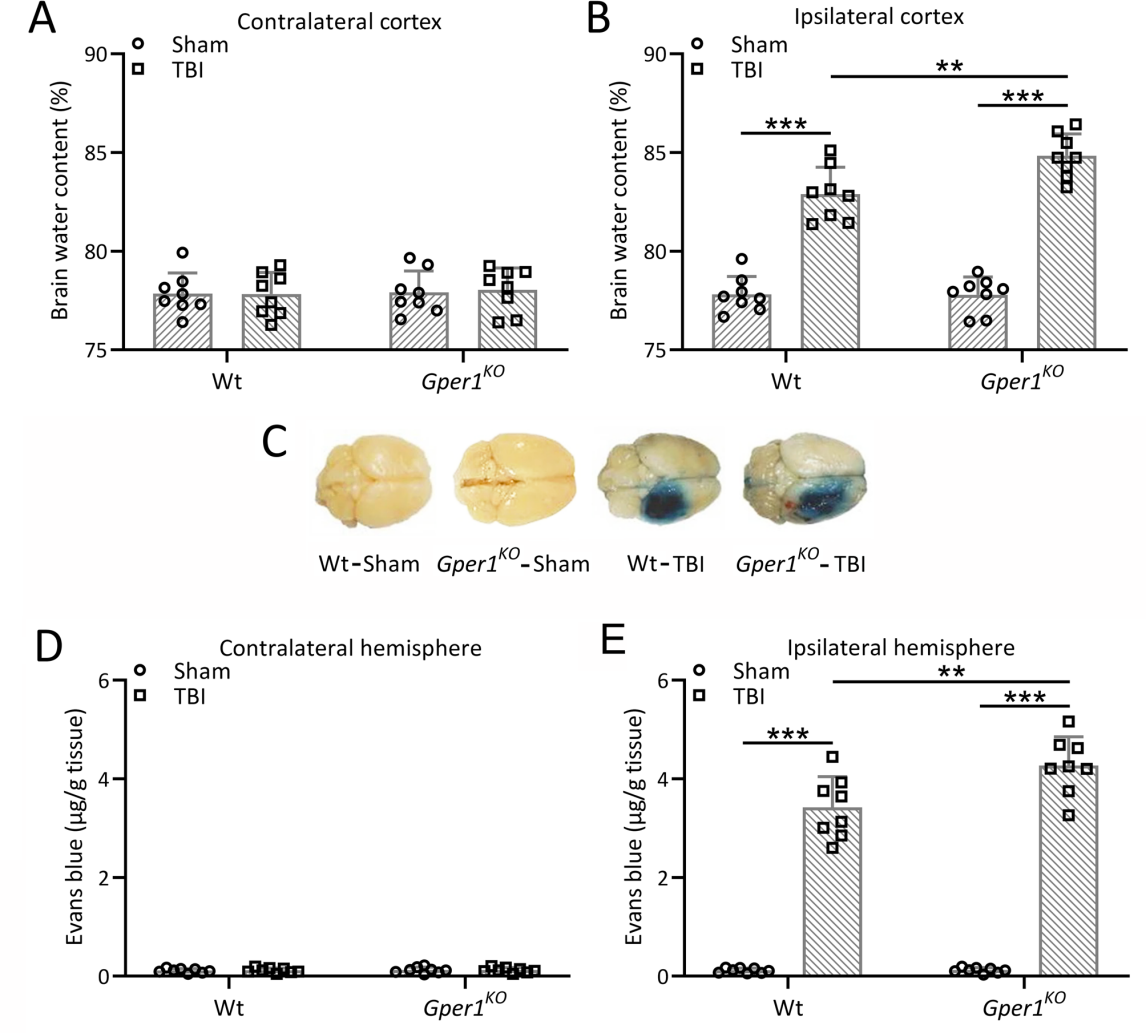
Gper1 knockout exacerbated traumatic brain injury induced brain edema and brain evans-blue extravasation. *Gper1*^KO^and wild type mice were applied to TBI. Brain water contents at contralateral cortex (**A**) and injury ipsilateral cortex (**B**) were compared 3 days after TBI. Evans blue extravasation was used to measure the blood-brain barrier permeability 3 days after TBI. (**C**) Representative images of Evans blue extravasation from each group. The contents of Evans blue between un-injury contralateral hemisphere (**D**) and injury ipsilateral hemisphere (**E**) were calculated in each group. 8 mice were used for each group. Data was shown with mean ± SD. **p < 0.01 and ***p < 0.001 from Two-way ANOVA followed Tukey’s multiple comparisons test

To further illustrate the potential protective effects of Gper1 on TBI, *Gper1* was knocked out in mice. To confirm the successful knockout of *Gper1* in *Gper1*^KO^ mice, both qRT-PCR and Western blot analyses were performed on cortical tissues from wild-type (WT) and *Gper1*^KO^mice. qRT-PCR results showed that the mRNA expression of Gper1 was completely absent in *Gper1*^KO^mice compared to WT mice (Figure S2A). Similarly, Western blot analysis demonstrated that GPER1 protein was undetectable in the cortex of *Gper1*^KO^mice, while it was clearly expressed in WT mice (Figure S2B and S2C). Brain water contents at contralateral cortex (Fig. 2A) and injury ipsilateral cortex (Fig. 2B) were compared 3 days after TBI. There was no significant difference between the water content of the contralateral cortex after TBI modeling. Cerebral edema was increased in the injury ipsilateral cortex of both wild-type and *Gper1*^*KO*^ mice after TBI modeling. However, brain water content was significantly higher in *Gper1*^*KO*^ mice than that in the wild-type mice, suggesting a protective role of Gper1 against TBI-induced brain edema. BBB damage caused by TBI was further analyzed. As shown in Fig. 2C and D, in the contralateral cortex of wild-type and *Gper1*^*KO*^ mice, the BBB was not significantly damaged before and after modeling. As shown in Fig. 2C and E, TBI resulted in obvious BBB impairment, and knockdown of *Gper1* significantly exacerbated BBB dysfunction in *Gper1*^*KO*^ mice.

### Gper1 knockout exacerbated TBI-induced cognitive impairment

**Fig. 3:**
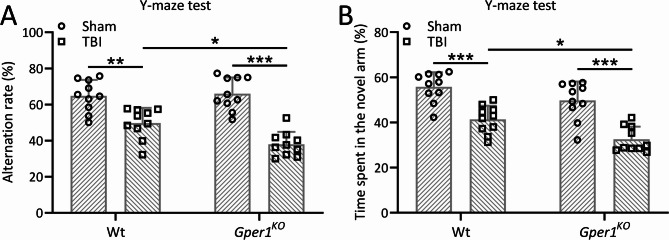
Gper1 knockout exacerbated traumatic brain injury induced cognitive impairments. *Gper1*^KO^and wild type mice were applied to TBI. Y-maze test was carried out at 8 days post-TBI. Spontaneous alternation (**A**) and time spent in the novel arm (**B**) were recorded. 10 mice were used for each group. Data was shown with mean ± SD. *p < 0.05, **p < 0.01, ***p < 0.001 from Two-way ANOVA followed Tukey’s multiple comparisons test

To analyze the protective effect of Gper1 on the cognitive function of TBI mice, Y-maze test was performed on wild-type mice and *Gper1*^*KO*^ mice on the 8th day after TBI modeling. The Y-maze test revealed that TBI significantly reduced spontaneous alternation behavior, indicating impaired spatial working memory. This reduction was more pronounced in *Gper1*^*KO*^ mice compared to wild-type mice (Fig. 3A). Additionally, the time spent in the novel arm was significantly reduced in TBI mice, with *Gper1*^*KO*^ mice showing a further decline compared to wild-type mice (Fig. 3B).

### Gper1 knockout exacerbated TBI-induced anxiety-like behaviors

**Fig. 4:**
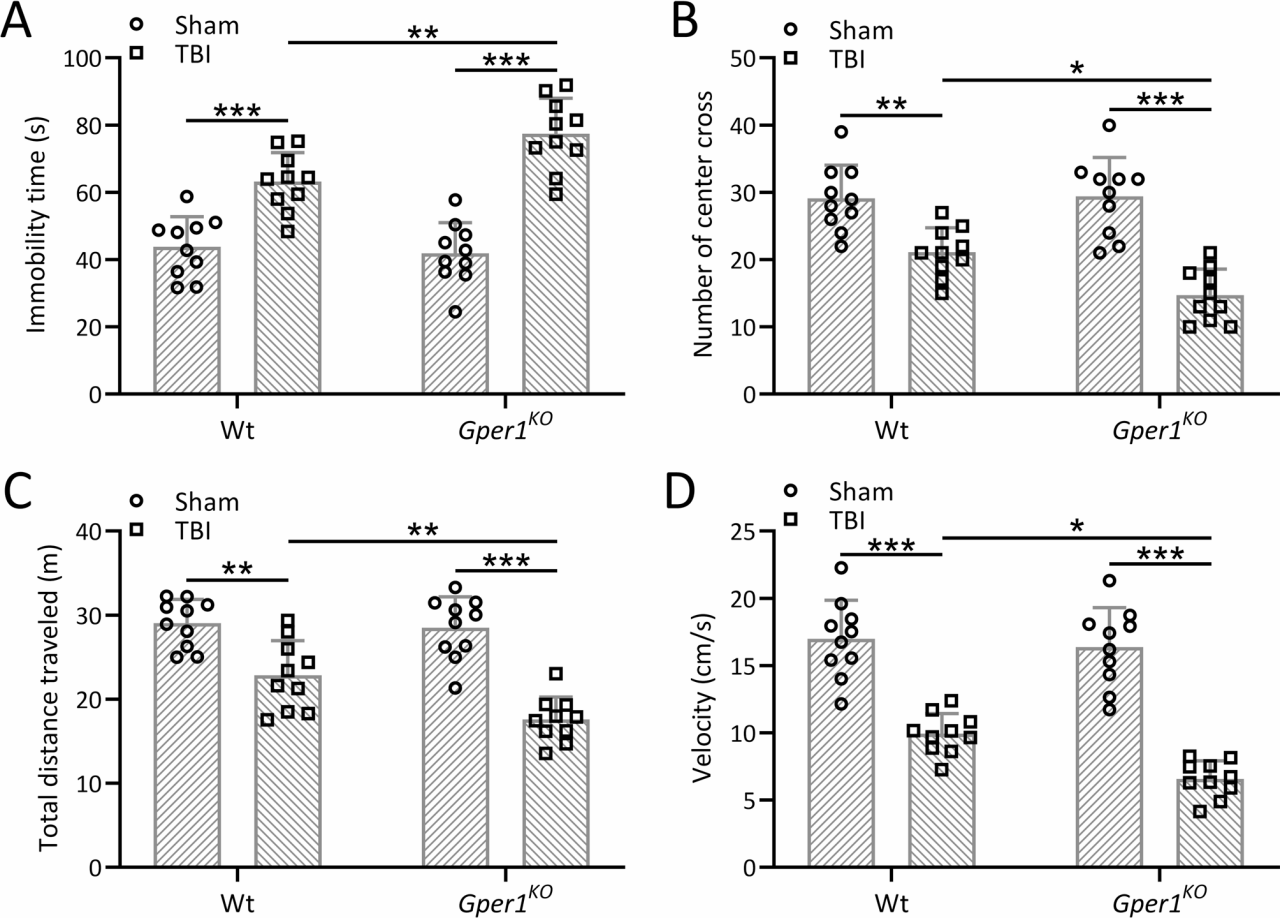
Gper1 knockout exacerbated traumatic brain injury induced anxiety-like behaviors. *Gper1*^KO^and wild type mice were applied to TBI. Gper1 knockout increased immobility time (**A**), decreased the number of center cross (**B**) and the total distance traveled (**C**) and also the velocity (**D**) in the open-field test at 7 days post-TBI. 10 mice were used for each group. Data was shown with mean ± SD. *p < 0.05, **p < 0.01, ***p < 0.001 from Two-way ANOVA followed Tukey’s multiple comparisons test

To investigate the effect of Gper1 on anxiety-like behaviors in TBI mice, the open field test was conducted on wild-type and *Gper1*^*KO*^ mice on the seventh day post-TBI modeling. *Gper1*^*KO*^ mice exhibited a significant increase in immobility time compared to wild-type mice (Fig. 4A), indicating reduced exploratory activity. Furthermore, *Gper1*^*KO*^ mice displayed a marked decrease in the number of central crossings (Fig. 4B) and total distance traveled (Fig. 4C), as well as a reduction in average speed (Fig. 4D). To further explore anxiety-like behavior, the distance traveled in the central zone was analyzed and is presented in Figure S3. *Gper1*^*KO*^ mice showed a significantly reduced distance in the central zone compared to wild-type mice, reflecting increased avoidance of the center and a preference for the periphery (corners/walls). This parameter, combined with the decreased number of central crossings (Fig. 4B), provides indirect evidence of heightened anxiety-like behavior in *Gper1*^*KO*^ mice. These findings collectively suggest that Gper1 knockout exacerbates TBI-induced anxiety-like behaviors, likely through alterations in locomotor and exploratory activity and increased aversion to open spaces.

### Gper1 knockout exacerbated TBI-induced cell apoptosis in the ipsilateral cortex

**Fig. 5:**
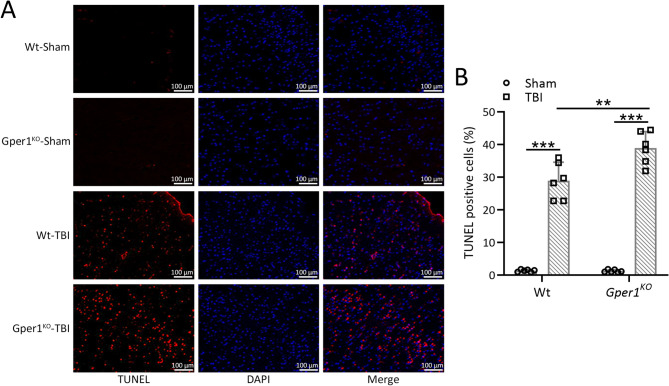
Gper1 knockout exacerbated traumatic brain injury induced cell apoptosis in the ipsilateral cortex. *Gper1*^KO^and wild type mice were applied to TBI. (**A**) Representative TUNEL staining of the lesioned cortices tissues in different groups and (**B**) the ratios of TUNEL positive cells at 7 days post-TBI. Scale bar, 10 μm. 6 mice in each group. Data was shown with mean ± SD. **p < 0.01, ***p < 0.001 from Two-way ANOVA followed Tukey’s multiple comparisons test

To demonstrate the protective effects of Gper1 on mouse brain cells, TUNEL staining was used to identify cell apoptosis in the cortical tissues of TBI injury on the seventh day after TBI. As shown in Fig. 5A and B, TBI modeling resulted in a marked increase in cell apoptosis in both wild type and *Gper1*^*KO*^ mice, whereas knockout of *Gper1* exacerbated TBI-induced cortical cell apoptosis.

### Gper1 knockout exacerbated TBI-induced neuroinflammation in the ipsilateral cortex

**Fig. 6:**
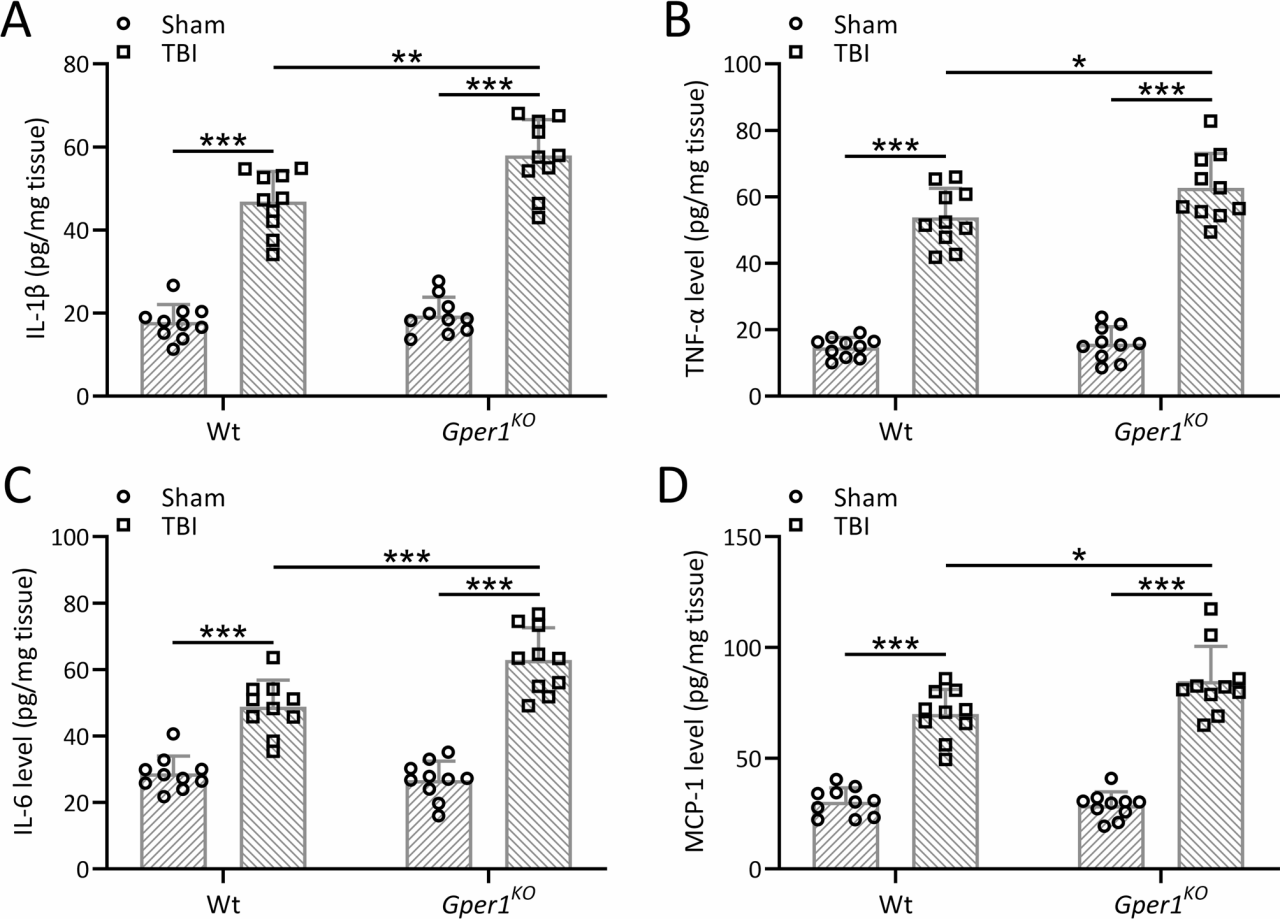
Gper1 knockout exacerbated traumatic brain injury induced neuroinflammation in the ipsilateral cortex. *Gper1*^KO^ and wild type mice were applied to TBI. Protein levels of IL-1β (**A**), TNF-α (**B**), IL-6 (**C**) and MCP-1 (**D**) in the lesioned cortices were measured by ELISA at 9 days post-TBI. 10 mice were used for each group. Data was shown with mean ± SD. *p < 0.05, **p < 0.01, ***p < 0.001 from Two-way ANOVA followed Tukey’s multiple comparisons test

In order to demonstrate the protective effects of Gper1 against the neuroinflammation induced by TBI, the protein levels of IL-1β (Fig. 6A), TNF-α (Fig. 6B), IL-6 (Fig. 6C) and MCP-1 (Fig. 6D) in the lesioned cortices were analyzed in both wild type and *Gper1*^*KO*^ mice. The levels of IL-1β, TNF-α, IL-6 and MCP-1 were all upregulated in the lesioned cortices in both wild type and *Gper1*^*KO*^ mice post TBI. However, the knockdown of Gper1 induced the further increase of IL-1β, TNF-α, IL-6 and MCP-1 in *Gper1*^*KO*^ mice compared with wild type mice, suggesting the protective role of Gper1 in the neuroinflammation caused by TBI.

### Gper1 knockout exacerbated traumatic brain injury induced activation of NLRP3 in the ipsilateral cortex

**Fig. 7:**
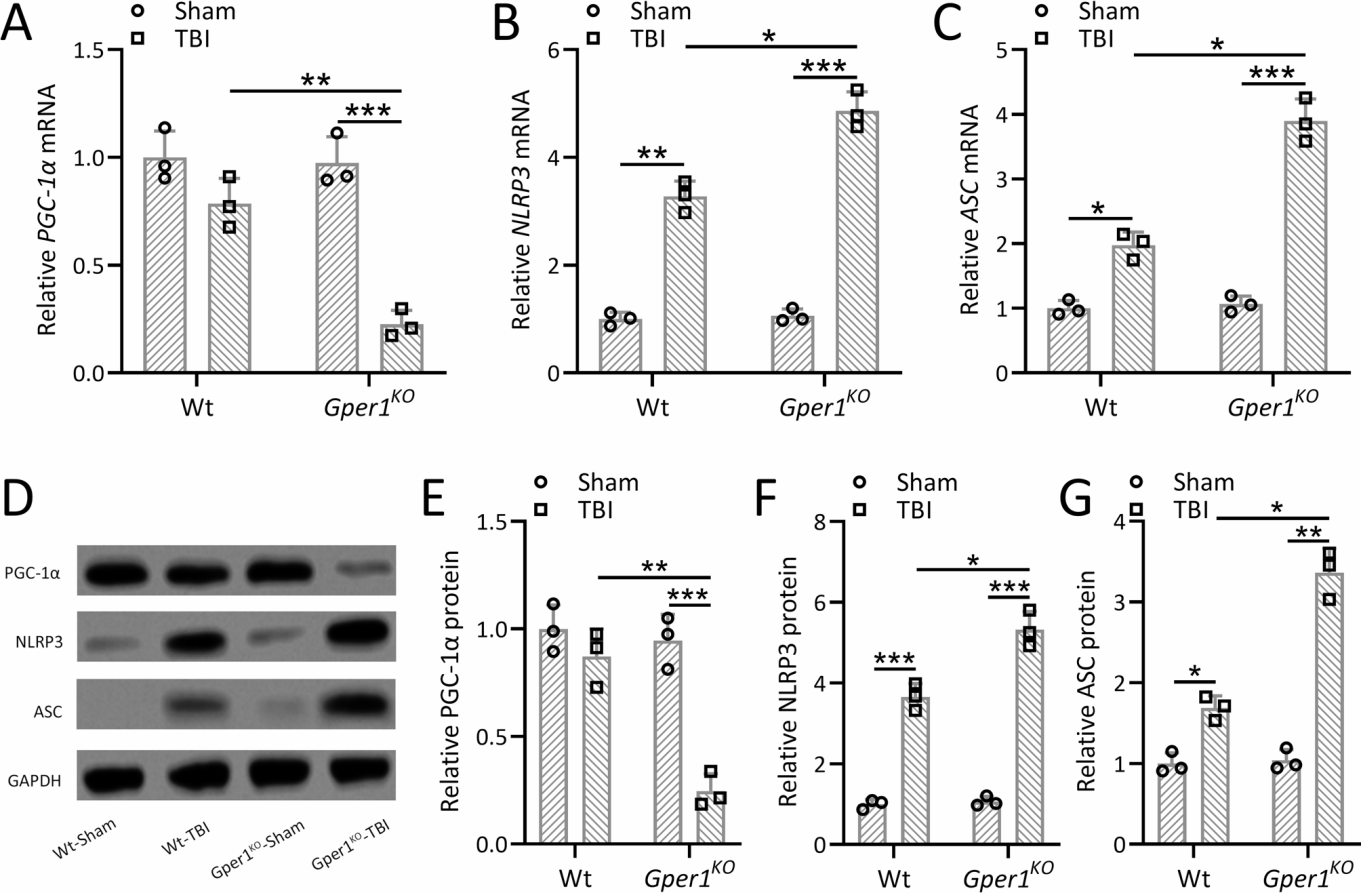
Gper1 knockout exacerbated traumatic brain injury induced activation of NLRP3 in the ipsilateral cortex. *Gper1*^KO^, and wild type mice were applied to TBI. qRT-PCR was used to measure the mRNA expressions of PGC-1α (**A**), NLRP3 (**B**) and ASC (**C**) in the lesioned cortices at 9 days post-TBI. (**D**), Western blotting was used to measure the protein expressions of PGC-1α, NLRP3 and ASC in the lesioned cortices at 9 days post-TBI. GAPDH was used as a loading control and the expressions were normalized to Wt-Sham (**E-G**). *N* = 3 repeats for each group (10 tissue homogenates were mixed for each group). Data was shown with mean ± SD. *p < 0.05, **p < 0.01, ***p < 0.001 from Two-way ANOVA followed Tukey’s multiple comparisons test

To investigate the impact of Gper1 knockout on TBI-induced inflammatory responses, mRNA and protein levels of PGC-1α, NLRP3, and ASC were measured in the lesioned cortices of *Gper1*^*KO*^ and wild-type (WT) mice at 9 days post-TBI. Quantitative RT-PCR revealed significantly reduced mRNA expression of PGC-1α in *Gper1*^*KO*^ mice compared to WT mice, while the mRNA levels of NLRP3 and ASC were markedly increased (Fig. 7A-C). Consistently, Western blot analysis demonstrated that protein expression levels of PGC-1α were decreased, whereas NLRP3 and ASC were significantly elevated in the lesioned cortices of *Gper1*^*KO*^ mice compared to WT mice (Fig. 7D-G). GAPDH was used as a loading control, and protein expressions were normalized to the WT-Sham group. These findings indicate that the absence of Gper1 enhances the activation of NLRP3 inflammasome components following TBI.

## Discussion

TBI is a common acute disease with poor prognosis and high mortality [14]. TBI-induced tissue hemorrhage and inflammatory response lead to insufficient oxygen supply in brain tissue and necrosis of nerve cells, which generally lead to various neurological sequelae in TBI patients [15]. TBI is a complex pathological and physiological change involving various immune cells and immune factors [16]. The central links in the development of TBI are brain cell ischemia, hypoxia and reperfusion. During the reperfusion process, a large number of oxygen free radicals are produced, which damage the nerve cell membrane and mitochondria. Metabolism and microcirculation disorders occur in the brain tissue of TBI patients, resulting in brain tissue ischemia, leukocyte infiltration and inflammatory response [17]. TBI can also lead to blockage of aerobic metabolism and increase of anaerobic glycolysis in brain tissue, causing a large influx of calcium ions in nerve cells and inducing a “waterfall” inflammatory mediator response [18]. The increase of oxygen free radicals and the toxic effects of excitatory amino acids lead to nerve cell damage and death.

In-depth research at the level of molecular biology helps us understand the pathological mechanism of disease occurrence and development, and has a great guiding role in clinical treatment. Previous literatures have revealed a large number of proteins that fight against inflammatory injury during the development of TBI, such as hypoxia inducible factor-1 (HIF-1), glucose transporter-1 (GLUT-1), small ubiquitin like-1 (SUMO-1) and CC chemokine ligand2 (CCL2), transient receptor potential cat-ion channel 1 (TRPC1) [19–22]. However, the signaling between neurons and the regulation of inflammation during TBI are multiple and complex. It is necessary to explore the interaction between neurotransmitters, inflammatory responses and signaling pathways, and then explain the occurrence and development of diseases more macroscopically, so as to provide accurate assessments of disease progression and prognosis for patients. In this study, we also further revealed the protective effect of a new TBI protective factor Gper1 on brain cell function and brain tissue structure during the occurrence and development of TBI.

GPER1 is an estrogen membrane receptor that mediates estrogen nongenomic biological effects in neurons [23]. GPER1 is a class of 7 transmembrane receptors consisting of 375 amino acids [24]. Its gene is located on chromosome 7p22 and contains a total of 7008 base pairs [25]. As a membrane receptor for estrogen, GPER1 is different from conventional nuclear receptors, and traditional nuclear receptor blockers cannot block its biological effects. Filardo et al. found that estrogen can activate the activity of Erk1/2 in breast cancer 4 cells in breast cancer cells that only express GPER1 and do not express estrogen nuclear receptors, which makes the relationship between GPR30 and estrogen begin to be paid attention to [26]. After the estrogen membrane receptor GPER1 was discovered, its biological effects in the nervous system aroused the interest of many scholars. Matsuda et al. observed that GPER1 protein and mRNA were significantly expressed in pyramidal cells in the CA1-3 region of the hippocampus and granule cells in the dentate gyrus [24]. In addition, our previous research found that the expression of GPER1 in the hippocampus changed. The expression of GPER1 began to increase after 6 h of ischemia, reached a peak at 48 h, and began to decrease at 72 h after global cerebral ischemia in rats [13]. In recent years, some studies have found that GPER1 not only exists in the brain, but also exerts physiological functions [27]. For example, Diane Lebesgue et al. found that the GPER1 agonist G1 can increase excitatory postsynaptic currents in vertebral cells in hippocampal slices of ovariectomized female rats [28]. It has also been reported that GPER1 can promote the release of calcium in neurons and activate the MAPK signaling pathway [29].

Our previous work found that the GPER1 agonist G1 can significantly reduce brain edema and alleviate TBI-induced cognitive impairment in mice. Activation of GPER1 regulates microglia-mediated neuroinflammation. This study continues to unearth the role and mechanism of GPER1 in TBI. We constructed TBI models on GPER1 conditional knockout mice and wild type mice, and studied the effects of GPER1 knockout on brain edema, cognitive function, neuro-apoptosis and inflammation in TBI mice. Similar to previous studies, we confirmed that TBI resulted in increased expression of Gper1 in mouse brain tissue. Similar to previous studies, we confirmed that TBI resulted in increased expression of Gper1 in mouse brain tissue. Our study showed that Gper1 knockout exacerbated TBI-induced cognitive dysfunction, and increased anxiety in mice. Gper1 knockdown also leads to exacerbation of TBI-induced neuro-apoptosis and inflammatory response.

Neuroinflammation, a hallmark of TBI, was markedly aggravated in *Gper1*^*KO*^ mice, as evidenced by elevated levels of pro-inflammatory cytokines (IL-1β, TNF-α, IL-6, and MCP-1) and increased activation of the NLRP3 inflammasome. These findings underscore the anti-inflammatory role of GPER1, possibly mediated through its regulation of microglial activation. Reduced expression of PGC-1α in *Gper1*^*KO*^ mice further supports the notion that GPER1 modulates mitochondrial function and energy metabolism during the inflammatory response.

TBI-induced neuronal apoptosis was also exacerbated in *Gper1*^*KO*^ mice, as demonstrated by increased TUNEL-positive cells in the cortex. GPER1’s neuroprotective effects may involve downstream signaling pathways, such as MAPK and calcium release, which have been shown to promote cell survival. Behavioral assessments revealed that *Gper1*^*KO*^ mice displayed heightened anxiety-like behaviors and impaired spatial working memory, further emphasizing GPER1’s critical role in preserving cognitive and emotional function after TBI.

To address the mechanistic underpinnings of GPER1’s protective effects in TBI, future research will focus on elucidating upstream regulators and downstream effectors of GPER1 by leveraging publicly available transcriptomic and proteomic datasets. Integrative bioinformatics approaches, such as co-expression network analysis and pathway enrichment tools, will be employed to identify key molecular interactions and signaling pathways modulated by GPER1. Additionally, causality can be further probed through systems biology frameworks that combine time-series gene expression data with regulatory network modeling, potentially uncovering candidate transcription factors or signaling intermediates that act upstream of GPER1 or mediate its downstream neuroprotective functions. These in silico predictions will guide experimental validations, such as CRISPR-based perturbations or pharmacological modulation in relevant cellular and in vivo models. Ultimately, these efforts aim to delineate the broader regulatory landscape of GPER1 and enhance our understanding of its role in mitigating TBI-induced pathologies.

While this study provides substantial evidence of GPER1’s protective effects, several limitations should be noted. First, only female mice were used, limiting the generalizability of the findings to male subjects. The influence of sex-specific hormonal differences on GPER1 function warrants further investigation. Second, the study primarily focuses on acute and subacute phases of TBI; long-term effects of *Gper1* knockout on neuroinflammation and cognitive recovery remain unexplored. Third, while the use of *Gper1*^*KO*^ mice allowed us to delineate GPER1’s role, pharmacological modulation of GPER1 could provide additional insights into its therapeutic potential. Last, only a single mouse TBI model was employed, validation in other TBI models, other species or in vitro systems should be conducted in the future.

## Conclusions

Our results demonstrate that Gper1 is involved in TBI-induced neuro-apoptosis, neuroinflammation and cognitive impairments in mice. The current findings suggest that Gper1 may be a therapeutic target for the treatment of TBI.

## Electronic supplementary material

Below is the link to the electronic supplementary material.

Supplementary Material 1

## Data Availability

No datasets were generated or analysed during the current study.
