# Supplementary material for: Gper1 inhibition exacerbates traumatic brain injury-induced neurological impairments in mice
Source: Behav Brain Funct. 2025 Jul 2;21:19. doi: 10.1186/s12993-025-00281-2 (PMC12224363; doi:10.1186/s12993-025-00281-2)
Supplement: Supplementary file 1 — Supplementary Material 1 [file 12993_2025_281_MOESM1_ESM.docx]

Supplementary materials


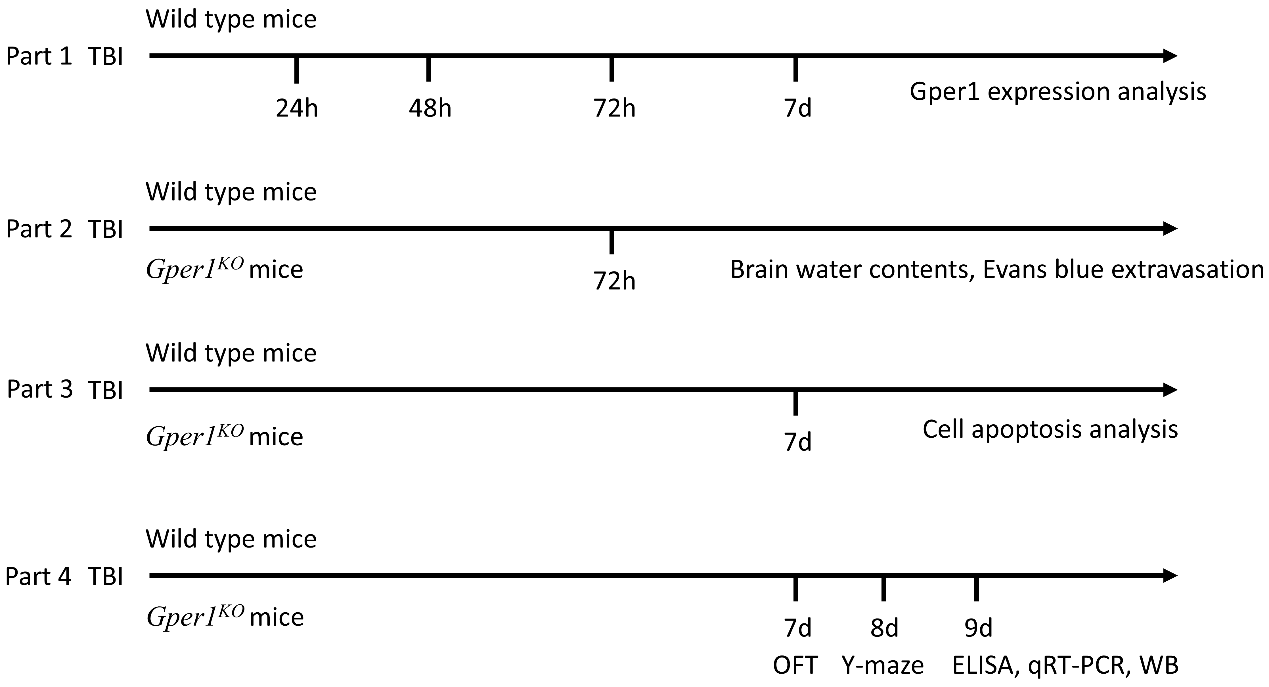


Figure S1. Experiment flow chart of the study.


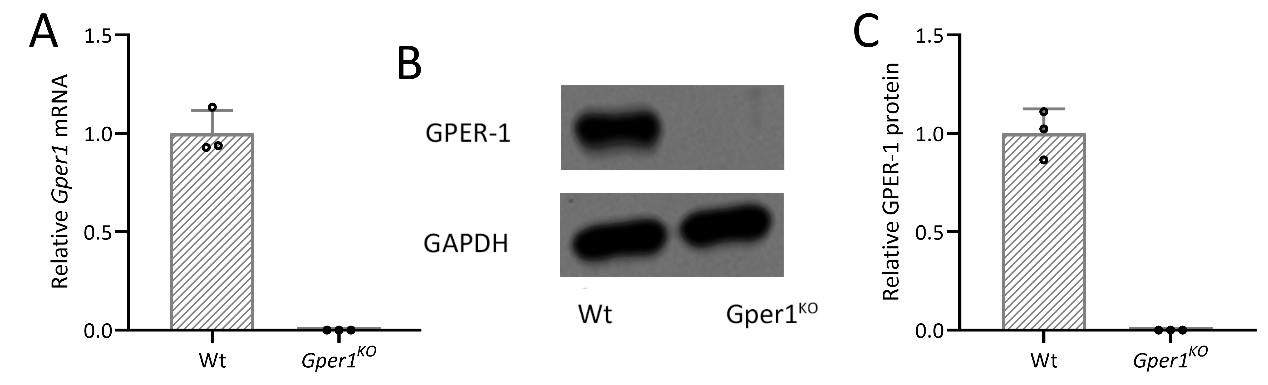


Figure S2. Identification of *Gper1* knockout mice. qRT-PCR and western blotting were used to measure the mRNA (A) and proteins (B and C) levels of Gper1 in the cortex from wild type or Gper1KO mice.


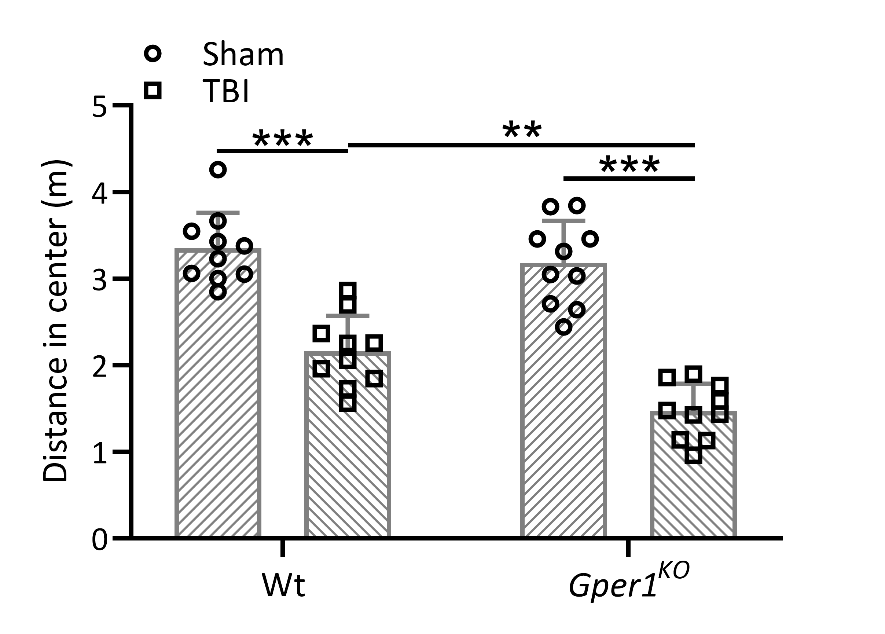


Figure S3. Comparison of distance in center in the OFT test. 10 mice were used for each group. Data was shown with mean ± SD. **p < 0.01, ***p < 0.001 from Two-way ANOVA followed Tukey's multiple comparisons test.
